# Reinforcement of Different Sands by Low-pH Bio-Mineralization

**DOI:** 10.3390/ma16186211

**Published:** 2023-09-14

**Authors:** Yongming Lai, Shiyu Liu, Yanyan Cai, Jin Yu

**Affiliations:** 1 College of Civil Engineering, Huaqiao University, Xiamen 361021, China; lym@lyun.edu.cn (Y.L.);; 2 College of Resource Engineering, Longyan University, Longyan 364000, China

**Keywords:** low pH, one-phase method, MICP, bioflocculation lag period, properties of sand

## Abstract

Different sands have significant influences on MICP reinforcement effects. Using calcium carbonate production and bioflocculation lag period as evaluation criteria, this study investigates the optimal theoretical pH values of bacterial solutions with different concentrations. We reinforced four different sands using MICP at the optimal theoretical pH, and based on permeability, moisture retention, raindrop erosion, wind erosion, penetration, and SEM tests, the influence of sand properties on low-pH MICP reinforcement was analyzed and the low-pH MICP mechanism was revealed. The results indicate the following: (1) The optimal theoretical pH values for bacterial solutions with concentrations of 0.67 × 10^8^ cells/mL, 3 × 10^8^ cells/mL, and 10 × 10^8^ cells/mL are 4.5, 3, and 4, respectively. (2) With 0.67 × 10^8^, 3 × 10^8^, and 10 × 10^8^ cells/mL bacterial solutions, the strength of tailings sand containing calcium salt was 21.15%, 44.42%, and 13.61% higher than that of quartz sand, respectively. The effective reinforcement depth of alkaline reclaimed sand was 10, 8, and 6 mm lower than that of neutral calcareous sand, respectively. The strength of fine tailings sand was 70.41%, 58.04%, and 22.6% higher than that of coarse reclaimed sand. The effective reinforcement depth of fine quartz sand was 6, 4, and 4 mm lower than that of coarse calcareous sand. (3) Low pH temporarily suppresses urease activity, delaying calcium carbonate flocculation and enhancing reinforcement uniformity. To achieve optimal reinforcement effects, adjusting the actual optimal pH values of bacterial solution based on sand properties is essential in engineering applications.

## 1. Introduction

Microbially Induced Calcium Carbonate Precipitation (MICP) is a green and environmentally friendly technique for improving rock and soil media, effectively enhancing the mechanical properties of treated rock and soil materials [1,2]. The core principle of MICP involves utilizing microorganisms to decompose urea, resulting in the production of carbonate ions. Calcium carbonate precipitation is produced as these ions bind with calcium ions, thereby cementing soil particles and enhancing their liquefaction resistance, mechanical strength, water erosion resistance, and wind erosion resistance [3,4,5]. The reaction mechanism is illustrated in Equations (1)–(5) [6,7,8], where Equation (1) represents the overall reaction and Equations (2)–(5) depict the stepwise reactions. Among these, reactions (3)–(5) are reversible processes, where the concentration of reactants and pH values influence the equilibrium of these reversible reactions, subsequently affecting the generation of calcium carbonate precipitation [9].

(1) $${CO({NH}_{2})}_{2}+{Ca}^{2+}+2H_{2}O\overset{Ureolytic\;bacteria}{\to }{CaCO}_{3}↓+{2NH}_{4}^{+}$$

(2) $${CO({NH}_{2})}_{2}+H_{2}O\overset{Ureolytic\;bacteria}{\to }{NH}_{3}+H_{2}NCOOH$$

(3) $$H_{2}NCOOH↔{NH}_{3}+{CO}_{2}$$

(4) $${NH}_{3}+H_{2}O↔{NH}_{4}^{+}+{OH}^{-}$$

(5) $${CO}_{2}+H_{2}O↔H_{2}{CO}_{3}↔H^{+}+{HCO}_{3}^{-}↔{2H}^{+}+{CO}_{3}^{2-}$$

Two major challenges for applying MICP in large-scale practical engineering projects are clogging and complex procedures. As early as 1989, Stocks-Fischer et al. [10] reported severe flocculation and clogging near a grouting inlet when using a single injection of a mixture of bacterial solution and cementation solution. To address this issue, numerous researchers have attempted methodological improvements. Whiffin et al. [11] introduced the two-phase MICP injection method, which alleviates the flocculation and clogging near grouting inlets to some extent. Building upon the two-phase method, L. Cheng et al. [12] proposed a staged injection method, further reducing the localized accumulation of calcium carbonate crystals and enhancing the uniform distribution of calcium carbonate [13]. Harkes et al. [14] introduced a three-phase MICP injection method, resulting in a more uniform and stable distribution of bacterial cells as nucleation sites and calcium carbonate crystals. However, multi-step injection methods are often complex, and predicting the interactions between different injection solutions is challenging. To seek a simpler solution for clogging, L. Cheng et al. [15] further proposed a low-pH one-phase method. By injecting a mixture of low-pH bacterial solution and cementation solution in a single step, this method utilizes lower pH to delay the formation of calcium carbonate during the MICP process, mitigating clogging issues and simplifying procedures. Zhang et al. [16] compared the difference in reinforcement effects between the two-phase method and the low-pH one-phase method. The results show that the low-pH one-phase method performs better in three aspects: the uniformity of calcium carbonate distribution, the utilization rate of calcium ions in the reaction solution, and the unconfined compressive strength of the reinforced sample. Yang et al. [17] used CH_3_COOH instead of HCI to show that q CH_3_COOH-buffered low-pH one-phase injection method improves the uniformity of biocement treatment by creating a much longer lag period, which delays the biocementation process and allows the all-in-one solution to be distributed more uniformly in soil within the lag period.

The low-pH approach provides a theoretical possibility for the practical application of MICP in engineering projects. However, MICP is a complex bio-chemical reaction, and its process is influenced by various factors. Jamal Ahmad et al. [18] conducted research on various factors affecting the performance of MICP, among which chemical factors mainly include the carbon source, pH, type and concentration of the calcium source, and type and concentration of the bacteria. At the same time, the study also pointed out that the optimal nutrient concentrations for MICP treatment can vary depending on the bacterial species used, the type and concentration of the calcium source, and the environmental conditions. Nitish Kr Prajapati et al. [19] mentioned that several factors can affect MICP treatment used for soil reinforcement. These included the type of bacteria used, the concentration of bacteria, the concentration of cementation solution, and the pH and temperature of the environment. Xu et al. [20] selected sand samples of different particle sizes to conduct MICP injections. The results showed that different particle sizes had certain effects on the distribution uniformity of calcium carbonate and the final reinforcement strength. Appropriate particle size and reasonable gradation can improve the uniformity of reinforcement. Zhang et al. [21] discussed the effect of magnesium on the microbial reinforcement of sand and proved that a low molar ratio of Mg^2+^/Ca^2+^ increases the unconfined compressive strength and the strength reaches its maximum value when Mg^2+^/Ca^2+^ = 0.1/0.4. However, with an increase in the Mg^2+^/Ca^2+^ molar ratio, the precipitation of carbonate is inhibited and the strength is reduced. Multiple existing studies [22,23,24,25,26] indicate significant variations in reinforcement effects of sands with different properties. Besides the traditional focus on calcium carbonate precipitation, an important indicator for the low-pH approach is calcium carbonate flocculation lag period, which aims to address clogging issues. The pH and composition of different sands in actual engineering applications can differ significantly, potentially causing interference with injected low-pH bacterial solutions and affecting the reinforcement effects. Investigating suitable pH levels for different sands is of great significance for the application of the low-pH approach in practical engineering projects.

In this study, calcium carbonate production and bioflocculation lag period are used as evaluation criteria to investigate the theoretically best pH conditions for reactions with bacterial solutions at various concentrations. Four types of soil with varying particle sizes, pH, and compositions were selected to conduct reinforcement experiments based on the optimal theoretical pH. By combining the mechanical properties and microstructure of reinforced sands, the influence of sand properties on the effectiveness of the low-pH approach for sand reinforcement was thoroughly analyzed. This study provides insights into the mechanism and application conditions of the low-pH MICP approach for sand reinforcement.

## 2. Materials and Methods

### 2.1. Test Sands

To investigate the effect of different sand types on the effectiveness of low-pH MICP reinforcement, four types of sand were selected for this experiment: tailings sand, quartz sand, calcareous sand, and reclaimed sand. Among them, calcareous sand and reclaimed sand have larger particle sizes, while tailings sand and quartz sand have smaller particle sizes. Tailings sand and reclaimed sand are alkaline, while calcareous sand and quartz sand are nearly neutral. Reclaimed sand and quartz sand have a single composition, with over 90% being SiO_2_, whereas tailings sand and calcareous sand contain a significant amount of CaO.

**Figure 1:**
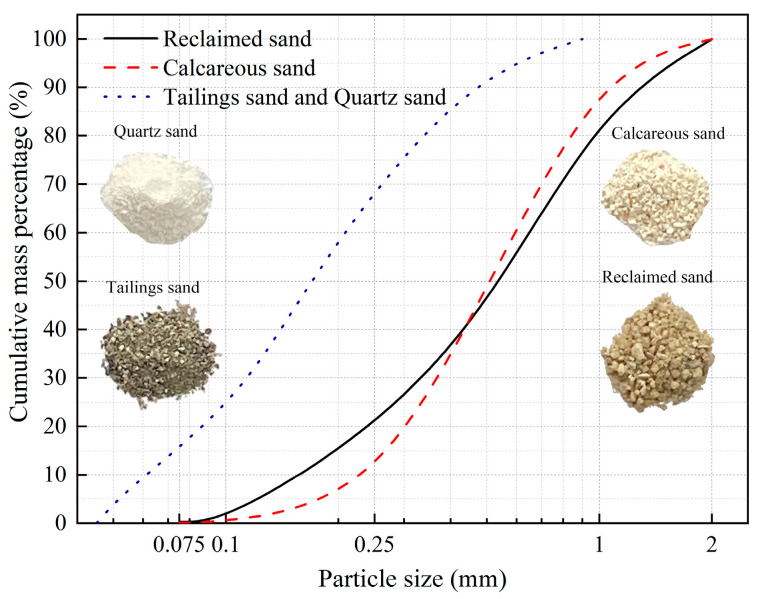
Particle size distribution curve and shape of test sands.

Tailings sand was sourced from the tailings dam of the Makeng Mine in Longyan, Fujian, China. Calcareous sand was collected from a reef in the South China Sea. reclaimed sand was obtained from the construction site of the Dadeng Reclamation Project in Xiamen, Fujian, China. A mixture of quartz sand with diverse particle sizes was prepared to match the particle distribution found in tailings sand. Figure 1 displays the particle size distribution curves and shapes for the four sand types. The minimum and maximum dry densities as well as pH of the four sands were determined according to Standard for Geotechnical Testing Method (GB/T 50123-2019) [27]. The main components of the four sands were analyzed using chemical analysis methods, and the results are shown in Table 1.

### 2.2. Bacterial Solution and Cementation Solution

Bacillus pasteurii (DSM33) was the selected bacterial strain for our experiments. The ATCC 1376 NH_4_-YE culture medium was utilized, containing yeast extract at 20 g/L, (NH_4_)_2_SO_4_ at 10 g/L, and Tris buffer at 15.75 g/L. The bacterial solution used in the experiments was collected from cultures in the stable phase after 48 h of cultivation. The absorbance of the bacterial solution was measured at a wavelength of 600 nm (OD_600_) using a spectrophotometer. At this point, the OD_600_ value was between 1.7 and 1.8. The bacterial concentration was calculated using Equation (6) [28], resulting in an approximate concentration of 1 × 10^8^ cells/mL. The bacterial solution exhibited a pH value of 8.5.

(6) $$Y=8.59\times 10^{7}Z^{1.3627}$$

where *Y* is the concentration of the bacterial solution (cells/mL) and *Z* is the OD_600_ value. The cementation solution consisted of a mixture of equimolar 2 mol/L CaCl_2_ and urea solutions. Sterilization was achieved through vacuum filtration.

### 2.3. Molds and Methods

Sterile culture dishes and disposable syringes were employed as molds for the experiments. The circular culture dishes, having a 9.5 cm diameter and a 1.6 cm height, accommodated a filling volume (V_1_) totaling 113.41 cm^3^. With dimensions of 3.29 cm in diameter and 12 cm in height, the disposable syringes possessed a filling height of 7 cm, giving rise to a filling volume (V_2_) totaling 60 cm^3^.

To prevent the influence of other bacteria, the four types of sand were dried in an oven at 120 °C for 12 h. Subsequently, they were individually filled into the two types of molds and compacted in three layers to achieve a filling density of 85% of the maximum dry density. At this point, the porosity of sand samples in the mold was 0.69 by calculation. A mixture of bacterial solution and cementation solution was evenly sprayed onto the surface of the sand samples using a spray bottle. The total sprayed volume of bacterial solution and cementation solution was set to 0.5 times the pore volume (0.5 eV), where e represents porosity and V represents volume of filled test sand in the mold. And the volume ratio between the bacterial solution and cementation solution was 1:1. For the culture dish sand samples, the total sprayed volume was approximately 40 mL (0.5 eV_1_), with 20 mL of both the bacterial solution and the cementation solution. For the syringe sand samples, the total sprayed volume was approximately 20 mL (0.5 eV_2_), with 10 mL of both the bacterial solution and the cementation solution. As a control group, an equal volume of deionized water was sprayed onto the surfaces of the sand samples. Experimentation for each group of sand samples was conducted in triplicate to reduce errors.

#### 2.3.1. Determination of the Optimal Theoretical pH Values

Our experiment involved the use of bacterial solutions at five different concentrations: 0.67 × 10^8^, 0.9 × 10^8^, 3 × 10^8^, 5 × 10^8^, and 10 × 10^8^ cells/mL, each adjusted to different pH values. For instance, the concentration of 0.67 × 10^8^ cells/mL was obtained by mixing 100 mL of pre-cultured bacterial solution with 50 mL of hydrochloric acid at varying concentrations. The 0.9 × 10^8^ cells/mL concentration was obtained by mixing 100 mL of pre-cultured bacterial solution with 10 mL of hydrochloric acid at varying concentrations. The three remaining bacterial solution concentrations were obtained via centrifugation. As an example, the 3 × 10^8^ cells/mL concentration was obtained by first centrifuging 50 mL of pre-cultured bacterial solution, discarding the supernatant, and then dissolving the resulting bacterial pellets in 16.7 (50/3) mL of sterile culture medium adjusted to the desired pH by adding an appropriate amount of hydrochloric acid during preparation. Similarly, the 5 × 10^8^ and 10 × 10^8^ cells/mL concentrations were obtained by dissolving the bacterial pellets in 10 (50/5) mL and 5 (50/10) mL of pre-adjusted sterile culture medium with different pH values.

Combining 50 mL of bacterial solution (with diverse concentrations and pH values) with 50 mL of 2 mol/L cementation solution was achieved in a beaker. The OD_600_ value of the top layer of the mixed solution was measured at regular intervals to calculate the amount of bacterial precipitation. After allowing the mixture to settle for 7 days, the pH of the completely reacted mixture was measured. The mixture was then filtered through filter paper, and two rounds of washing with anhydrous ethanol were carried out on the obtained precipitate. The amount of calcium carbonate generated was determined using a hydrochloric acid washing method [29].

Three different levels of bacterial solution concentration—low, medium, and high (0.67 × 10^8^, 3 × 10^8^, and 10 × 10^8^ cells/mL)—were selected for MICP spray treatment. The treated sand samples were left at room temperature (25 °C) for 7 days for complete reaction and then dried to a constant weight in a 60 °C drying oven. Subsequently, various performance tests were conducted.

#### 2.3.2. Permeability Test

The permeability coefficient of the sand samples in the syringes was determined using the constant head method according to GB/T 50123-2019 “Standard Test Methods for Soil Engineering”.

#### 2.3.3. Moisture Retention Test

The moisture retention test was conducted on dish sand samples. A record was made of the sand samples’ initial mass and then 20 g of deionized water was slowly and evenly poured into the sand samples. After the water was uniformly absorbed by the sand, the sand samples were placed in a 40 °C drying oven. The mass of the sand samples was recorded at regular intervals. The moisture content of the sand samples was calculated using the following Formula (7):

(7) $$\eta =(W_{i}-W_{0})/20\;*\;100\%$$

In this equation, η represents the sand sample’s moisture content (%), $W_{i}$ is the mass of the sand sample taken from the drying oven at different time intervals (g), $W_{0}$ represents the dried sand sample’s initial mass, and 20 is the mass of deionized water poured into the sand sample (g).

#### 2.3.4. Raindrop Erosion Test

**Figure 2:**
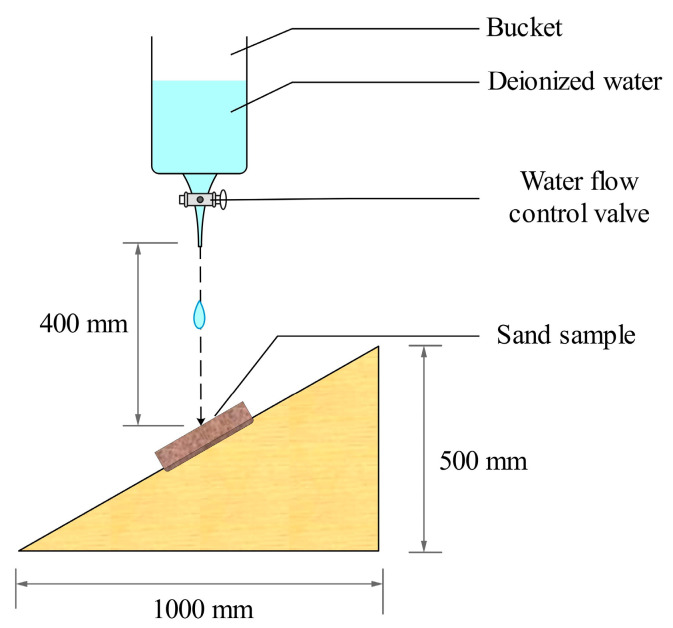
Raindrop erosion test.

Following the guidelines from the “Materials and Workmanship for Earth Buildings” (NZS 4298: 1998) standard [30], an inclined surface made of transparent acrylic was used to model raindrop erosion, with the dish sand samples positioned accordingly, having a length of 1 m, a height of 0.5 m, and a width of 0.15 m. Deionized water was released from a vertical position, falling from a distance of 40 cm above the sand sample’s center. The water droplet flow rate was adjusted to 3.33 mL/min, totaling 100 mL of water being dropped in 30 min (as shown in Figure 2). After the test, the erosion diameter (widest point) and erosion depth (deepest point) of the sand sample were measured using a vernier caliper. Subsequently, the sand sample was dried in a 60 °C drying oven to a constant weight. The erosion mass was calculated based on the initial mass of the sand sample before the test and the mass after drying.

#### 2.3.5. Penetration Resistance Test

**Figure 3:**
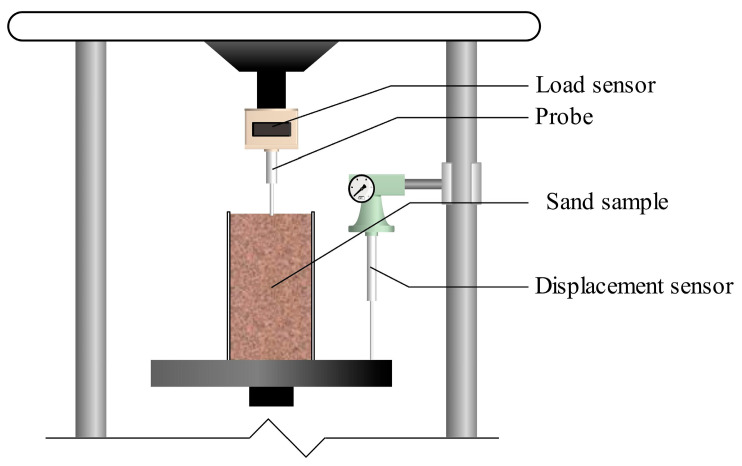
Penetration resistance test.

Referencing the method by M. A. Rice et al. [31] used to evaluate the abrasion resistance of crusts through a penetration resistance test, an improved micro-penetration device was used for the syringe sand samples (as shown in Figure 3) to simultaneously evaluate the reinforcement strength and uniformity. The improved micro-penetration device consists of a TSZ-3 strain-controlled triaxial tester, a load sensor (measuring range: 0~200 N), a probe (diameter: 3 mm), and a displacement sensor (measuring range: −25~25 mm). Before testing, the probe was placed directly above the center of the sand sample’s surface, and the strain rate was set at 4 mm/min. Both the penetration resistance and penetration depth of the probe were recorded with the load sensor and displacement sensor placed vertically. Data were recorded using the DH3816N static strain testing system.

#### 2.3.6. Wind Erosion Test

**Figure 4:**
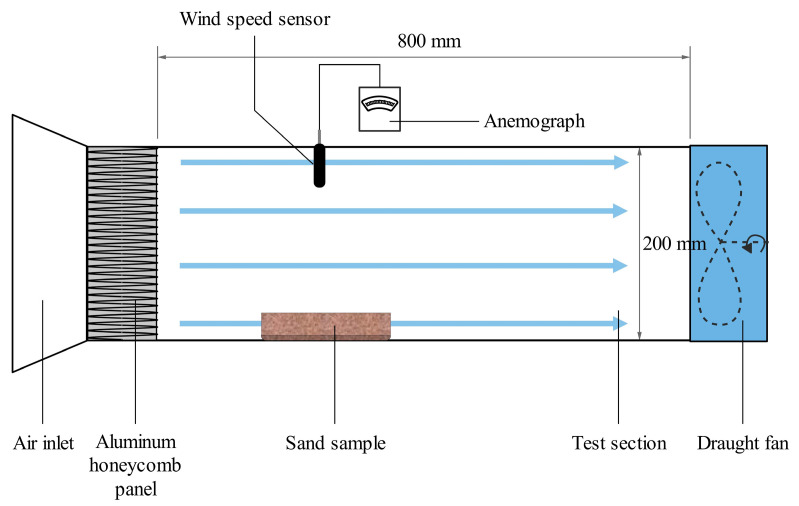
Wind erosion test.

Dish sand samples were placed in a small-scale wind tunnel model (as shown in Figure 4) and subjected to different wind speeds: 4, 6, 9, and 12 m/s. After 1 h of exposure, the mass loss due to wind erosion was measured for each sand sample.

## 3. Results

### 3.1. Determination of the Optimal Theoretical pH Values

**Figure 5:**
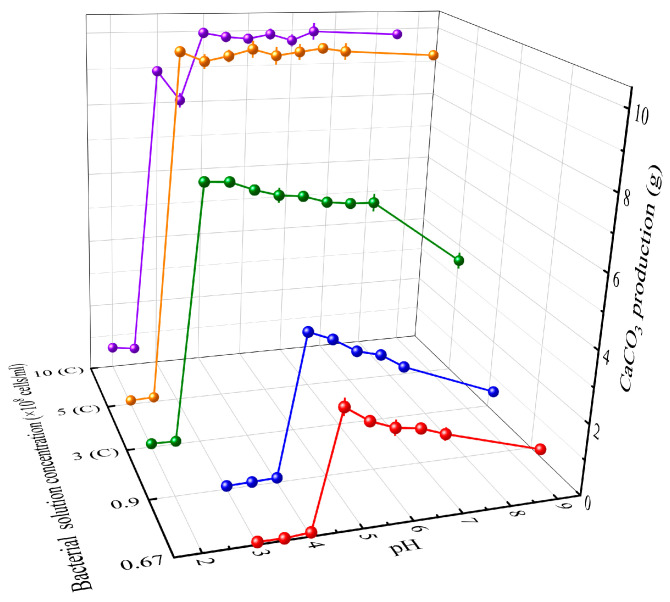
Calcium carbonate production (C represents that the bacterial solution is obtained from centrifugation).

**Figure 6:**
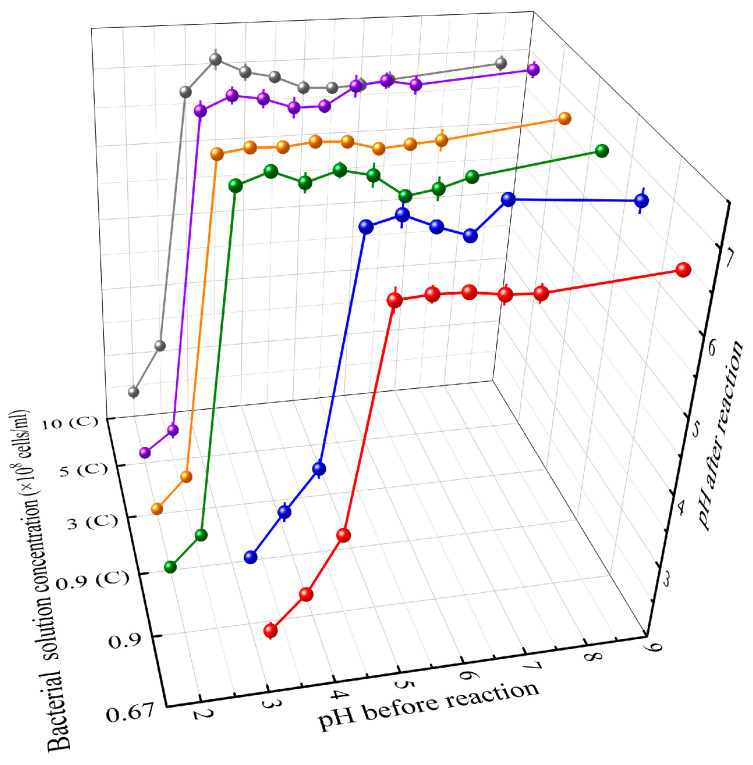
Changes in pH before and after reaction (C represents that the bacterial solution is obtained from centrifugation).

Figure 5 illustrates the calcium carbonate content generated after 7 days of reaction with bacterial solutions of different concentrations and pH levels and the cementation solution, with the original bacterial solution’s pH set at 8.5. The change in pH values of the bacterial solutions is graphically presented in Figure 6, encompassing the periods before and after the reaction.

In these figures, the concentrations are represented by 0.67, 0.9, 3, 5, and 10, corresponding to 0.67 × 10^8^, 0.9 × 10^8^, 3 × 10^8^, 5 × 10^8^, and 10 × 10^8^ cells/mL bacterial solution concentrations, respectively. It can be observed that both the calcium carbonate production and the post-reaction pH undergo a sudden change around a specific pH value. This specific pH is defined as the lowest tolerable pH value. When the pH of the bacterial solution drops below this lowest tolerable pH value, calcium carbonate precipitation is almost non-existent (Figure 5), and the pH of the bacterial solution remains nearly unchanged before and after the reaction (Figure 6). Because, at a lower pH (lower than pKa1 of carbonate system: 6.35), the chemical equilibrium of carbonate systems is displaced to H_2_CO_3_ and $HCO_{3}^{-}$ forms, it is very difficult to precipitate CaCO_3_. At this moment, the urease activity is very low and the amount of OH^−^ produced by decomposing urea is low, which has little effect on the pH of the carbonate system. So, urease gradually becomes completely inactive under long-term inhibition in a low-pH environment and the MICP process cannot proceed normally.

Furthermore, it is evident that various bacterial solution concentrations have different lowest tolerable pH values. The bacterial solutions obtained from centrifugation at 3 × 10^8^, 5 × 10^8^, and 10 × 10^8^ cells/mL exhibit a lowest tolerable pH of 3, while the two concentrations of 0.67 × 10^8^ and 0.9 × 10^8^ cells/mL have a lowest tolerable pH of 4.5. Considering that the bacterial solutions obtained from centrifugation (3 × 10^8^, 5 × 10^8^, 10 × 10^8^ cells/mL) are dissolved in freshly prepared culture medium, the bacteria have ample nutrients to withstand more acidic environments. On the other hand, the remaining two bacterial solution concentrations were cultured for 48 h, depleting the nutrients in the medium and resulting in reduced tolerance to acidic conditions. This inference is verified in Figure 6. For the same concentration of 0.9 × 10^8^ cells/mL, the bacterial solution obtained from centrifugation exhibits a lowest tolerable pH of 3, while the diluted bacterial solution has a lowest tolerable pH of 4.5. Therefore, the availability of abundant nutrients in freshly prepared culture medium is a key factor affecting pH tolerance, and pH tolerance is not directly related to bacterial solution concentration.

The MICP reaction mechanism can be described using Equations (1)–(5). Equation (1) represents the overall reaction, while Equations (2)–(5) represent the stepwise reactions. Equations (3)–(5) are reversible, and both the reactant concentration and pH values play a role in shifting the equilibrium of this reversible reaction, consequently impacting calcium carbonate precipitation.

In Figure 5, within the tolerable pH range of 0.67 × 10^8^, 0.9 × 10^8^, and 3 × 10^8^ cells/mL bacterial solution concentrations, a lower pH leads to a higher level of calcium carbonate production. This phenomenon is attributed to the disruption of the initial equilibrium of reversible reactions (3)–(5) in an acidic environment. With lower pH, the concentration of H^+^ increases. One of the products, OH^−^, reacts with H^+^ in the solution, reducing its concentration. This promotes the reversible reaction (4) to proceed in the forward direction, subsequently decreasing NH^3^ concentration and causing reversible reaction (3) to shift in the forward direction as well. During the process of neutralizing excess H^+^ with OH^−^, the reversible reactions (3)–(5) continue to progress in the forward direction until a new equilibrium is reached. This also explains why the solution’s pH value after the reaction is around 7 (Figure 6). Hence, when there is an ample supply of calcium sources, within the tolerable pH range, a lower pH encourages more forward progression of the reversible reactions (3)–(5), leading to more calcium carbonate production and more calcium carbonate precipitates are formed by combining with Ca^2+^.

For bacterial concentrations of 5 × 10^8^ and 10 × 10^8^ cells/mL, the generated calcium carbonate stabilizes at around 10 g. This occurs because, in these cases, there is an excess of bacteria but a limited supply of calcium sources. An amount of 50 mL of the 2 mol/L cementation solution contains 0.1 mol of Ca^2+^. Therefore, the maximum possible amount of generated calcium carbonate is 10 g (100 g/mol × 0.1 mol). It is important to note that for the 10 × 10^8^ cells/mL bacterial concentration at pH values of 3 and 3.5, the generated calcium carbonate amount is lower than 10 g. This is due to the extremely high bacterial concentration, and some bacteria may die due to insufficient nutrients to withstand the harsh acidic environment (pH: 3 and 3.5). The reduced bacterial concentration leads to a decrease in the induction of calcium carbonate production.

In Figure 6, for bacterial solutions within the tolerable pH range, the pH after the reaction stabilizes at around 7. This indicates that the reversible reactions (3)–(5) have reached an equilibrium state, confirming the earlier inference. On the other hand, for bacterial solutions with pH below the minimum tolerable level, the generated calcium carbonate amount is approximately 0 (Figure 5), and the pH remains relatively unchanged before and after the reaction (Figure 6). This signifies that bacterial urease is deactivated in these conditions, and the MICP process cannot proceed.

In a low-pH environment, the initial activity of bacterial urease is inhibited, leading to a reduction in urea decomposition and subsequent calcium carbonate precipitation. As the reaction progresses, the pH gradually increases, and the urease activity starts to recover. By periodically measuring the OD_600_ values of the supernatant after mixing bacterial solutions with the cementation solution, the suspended bacterial concentration can be determined, allowing the calculation of the percentage of flocculating bacteria. The time taken to achieve 30% flocculating bacteria is defined as the bioflocculation lag period. In the application of MICP engineering, this lag period with limited precipitation can be utilized to expand the penetration range, effectively enhancing reinforcement depth and uniformity.

**Figure 7:**
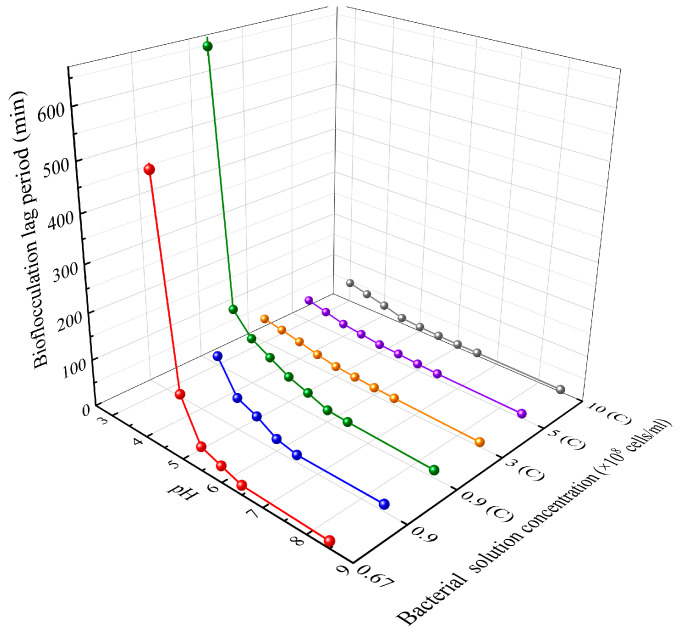
Bioflocculation lag period (C represents that the bacterial solution is obtained from centrifugation).

Figure 7 illustrates the bioflocculation lag period for different concentrations and pH levels of bacterial solutions mixed with the cementation solution. The length of the bioflocculation lag period depends on the rate of pH increase in the solution. For a given bacterial concentration, a lower pH leads to a slower increase in pH due to a higher concentration of H^+^, resulting in a longer bioflocculation lag period. On the other hand, for a given pH level, higher bacterial concentrations generate OH^−^ ions more rapidly, leading to a quicker increase in pH and a shorter bioflocculation lag period. Therefore, within the tolerable pH range, lower pH and bacterial concentrations result in longer bioflocculation lag periods, contributing to a more uniform MICP reinforcement effect.

### 3.2. Permeability Test

**Figure 8:**
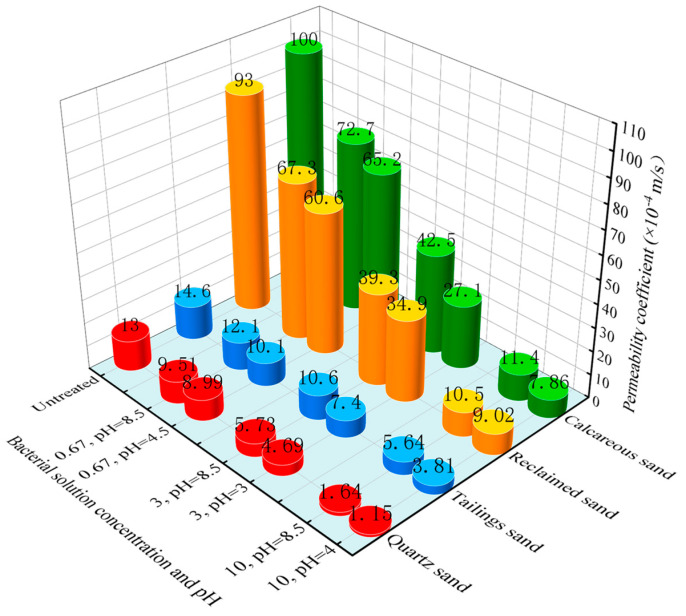
Permeability coefficient.

Figure 8 displays the changes in permeability coefficients for the four types of sands before and after MICP treatment. Prior to treatment, the permeability coefficients ranked as follows: calcareous sand > reclaimed sand > tailings sand > quartz sand. The first two are coarse sands, resulting in significantly higher permeability coefficients compared to the latter two fine sands. Calcareous sand contains fewer fine particles and more coarse particles, leading to a larger porosity compared to reclaimed sand, resulting in a higher permeability coefficient. Tailings sand, containing calcium oxide, is more susceptible to erosion by water, hence having a higher permeability coefficient than quartz sand.

After MICP treatment, the permeability coefficients of the sand samples were significantly reduced, with higher bacterial concentrations leading to more pronounced reductions. Furthermore, samples treated with low-pH bacterial solution exhibited more significant reductions in permeability coefficients compared to samples treated with pH-unadjusted bacterial solution. This is attributed to the fact that low-pH bacterial solution induces the formation of more and evenly distributed calcium carbonate, which improves the pore-filling effect and reduces permeability.

It is worth noting the substantial differences in the effect of the two treatment methods on calcareous and tailings sands. This could be attributed to the presence of calcium salt in these two sands, which provides additional calcium sources. Under the influence of the low-pH lag period, more effective calcium carbonate is generated, enhancing consolidation. At a bacterial concentration of 0.67 × 10^8^ cells/mL, the trends in permeability changes for the four sands after MICP treatment remained consistent with the pre-treatment characteristics. However, for bacterial concentrations of 3 × 10^8^ and 10 × 10^8^ cells/mL, the permeability coefficient of calcareous sand was lower than that of reclaimed sand after MICP treatment under low-pH conditions. This is due to the presence of additional calcium sources and the larger porosity of calcareous sand, resulting in deeper and more uniform permeation when bacterial concentrations are sufficient, leading to better calcium carbonate precipitation and uniformity compared to reclaimed sand. At a bacterial concentration of 0.67 × 10^8^ cells/mL, the effect was less pronounced due to insufficient bacterial concentration.

### 3.3. Moisture Retention Test

**Figure 9:**
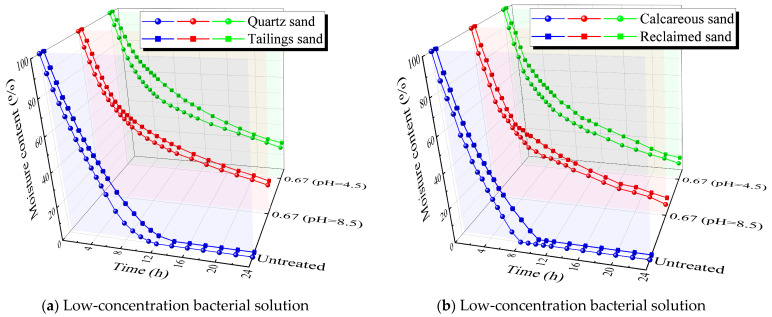

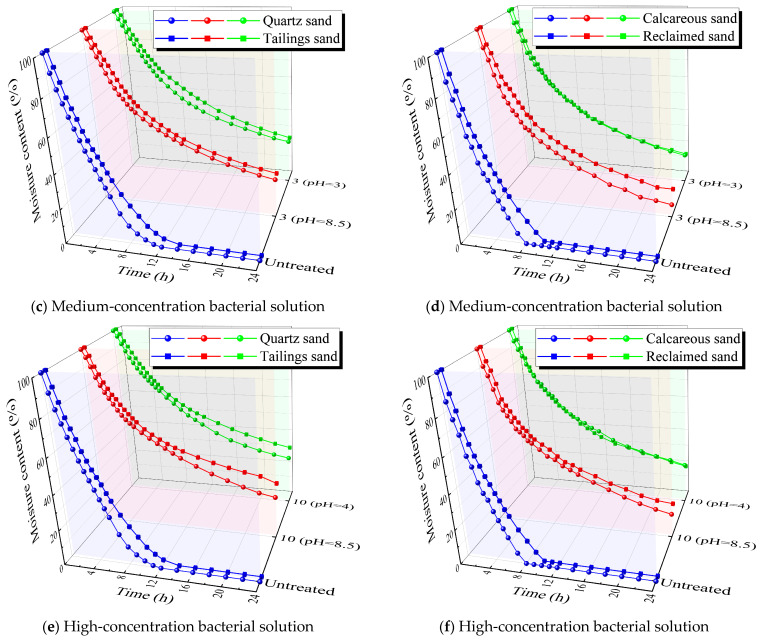
Moisture content.

Figure 9 illustrates the variation in moisture content over time before and after MICP treatment for the four types of sands. The initial moisture retention properties were ranked as follows: tailings sand > quartz sand > reclaimed sand > calcareous sand. The first two sands are fine-grained, characterized by small pores, resulting in significantly higher moisture retention compared to the coarse sands. Tailings sand contains calcium oxide, which imparts a certain water-absorbing capability, leading to higher moisture retention than quartz sand. calcareous sand contains fewer fine particles and more coarse particles, resulting in larger pores compared to reclaimed sand, thus exhibiting lower moisture retention.

After MICP treatment, the moisture retention properties of the sand samples were significantly enhanced, with higher bacterial concentrations resulting in better moisture retention. Similarly, samples treated with low-pH bacterial solution exhibited superior moisture retention compared to samples treated with pH-unadjusted bacterial solution. This phenomenon can be attributed to the greater quantity and more uniform distribution of calcium carbonate induced by the low-pH bacterial solution. At a bacterial concentration of 0.67 × 10^8^ cells/mL, the trends in moisture retention properties for the four sands after MICP treatment remained consistent with the pre-treatment characteristics. However, at bacterial concentrations of 3 × 10^8^ and 10 × 10^8^ cells/mL, the moisture retention properties of calcareous sand were higher than those of reclaimed sand after MICP treatment under low-pH conditions. This consistency aligns with the patterns observed in the permeability experiments.

### 3.4. Raindrop Erosion Test

**Figure 10:**
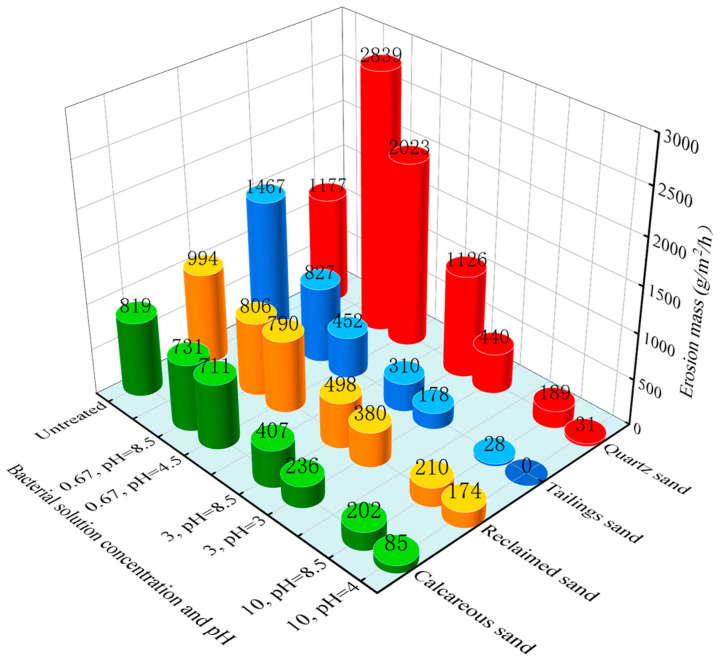
Raindrop erosion mass.

Figure 10 presents the variation in raindrop erosion mass per unit area over unit time for the four types of sands before and after MICP treatment. The initial erosion resistance was ranked as follows: tailings sand > quartz sand > reclaimed sand > calcareous sand. Smaller sand particles are more susceptible to erosion, and tailings sand, containing calcium oxide, is particularly vulnerable to erosive action by water, resulting in the highest erosion mass. Following MICP treatment, the erosion mass of the sand samples generally exhibited a significant reduction, with higher bacterial concentrations resulting in more pronounced decreases. Additionally, sand samples treated with low-pH bacterial solution experienced more pronounced erosion reduction compared to samples treated with pH-unadjusted bacterial solution. This is attributed to the greater quantity and more uniform distribution of calcium carbonate induced by the low-pH bacterial solution.

It is notable that, when it comes to quartz sand treated with a bacterial concentration of 0.67 × 10^8^ cells/mL, the erosion mass after MICP treatment exceeded that of the untreated quartz sand. This can be attributed to the low permeability of quartz sand and lower calcium carbonate production with low bacterial concentrations. As a result, only surface bonding of quartz sand particles occurred, and these bonded portions were more susceptible to being dislodged in the form of flakes during the erosion test.

Furthermore, the erosion mass of tailings sand treated with a low-pH bacterial solution at a concentration of 10 × 10^8^ cells/mL was reduced to zero, highlighting the superior raindrop erosion resistance of tailings sand among the four types of sands after reinforcement.

### 3.5. Penetration Resistance Test

**Figure 11:**
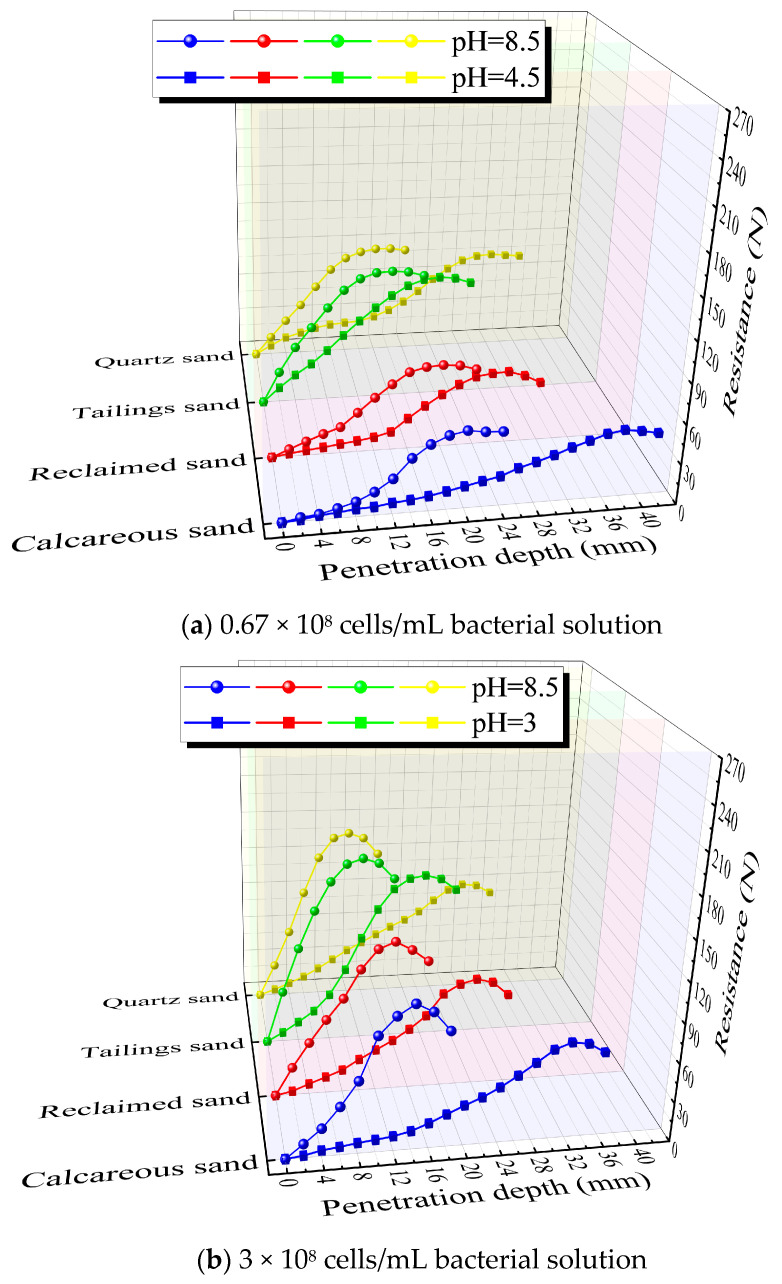

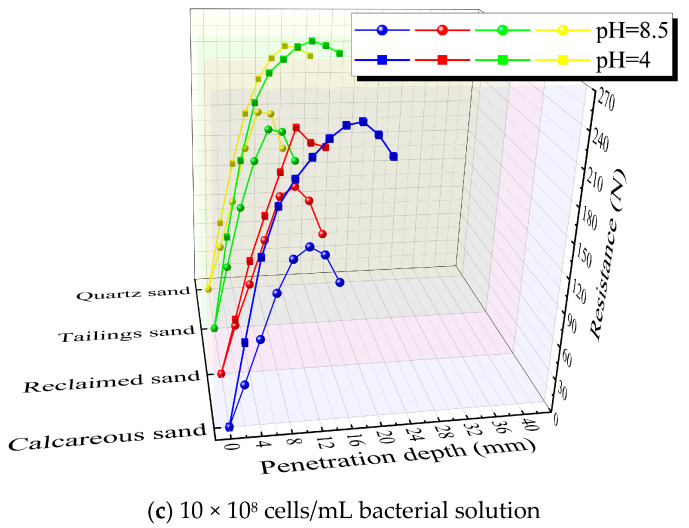
Variation in resistance with penetration depth.

Figure 11 depicts the variation in probe penetration resistance with penetration depth before and after the MICP treatment for the four types of sands. It can be observed that the penetration resistance of all sand samples initially increases and then decreases, with the depth corresponding to the peak strength representing the effective reinforcement depth. Following MICP treatment, all four types of sands exhibit certain strength improvements. For a specific type of sand, the effective reinforcement depth increases as the bacterial concentration decreases, while the strength exhibits an opposite trend. This phenomenon arises from the fact that lower bacterial concentrations result in longer bioflocculation lag periods, leading to deeper solution infiltration and, thus, greater effective reinforcement depths. However, the reduced calcium carbonate production at lower bacterial concentrations leads to decreased strength.

At concentrations of 3 × 10^8^ and 0.67 × 10^8^ cells/mL, the effective reinforcement depth achieved with low-pH bacterial solution is larger compared to that achieved with pH-unadjusted bacterial solution for the same type of sand. However, the strength is slightly lower. This discrepancy can be attributed to the extended bioflocculation lag period associated with low-pH bacterial solution, allowing for deeper and more uniform solution infiltration. On the other hand, the calcium carbonate induced by pH-unadjusted bacterial solution tends to accumulate locally, resulting in higher strength. For pH-unadjusted bacterial solution, the effective reinforcement depth ranks as calcareous sand ≈ reclaimed sand > tailings sand ≈ quartz sand. This is because pH-unadjusted bacterial solution has minimal bioflocculation lag period, and the effective reinforcement depth primarily relies on the permeability of sand samples. Coarser sands have larger permeability coefficients, allowing for deeper solution infiltration and, thus, greater effective consolidation depths. The strength after pH-unadjusted bacterial solution treatment ranks as tailings sand > quartz sand > reclaimed sand > calcareous sand. Smaller sand particles with smaller voids between them are more readily filled and connected by the generated calcium carbonate, resulting in denser samples and higher overall strength. Tailings sand contains calcium salt and heavy metal ions, which readily combine with carbonate ions to form effective cementitious compounds, thus yielding better reinforcement compared to quartz sand.

For low-pH bacterial solution treatment, the effective consolidation depth ranks as calcareous sand > quartz sand > reclaimed sand > tailings sand. This distinction is primarily driven by the inherent pH of the four sand types. Reclaimed sand and tailings sand exhibit alkaline properties, neutralizing the acidity introduced by the bacterial solution, causing an increase in pH. As a result, the bioflocculation lag period is shortened, leading to shallower solution infiltration. Additionally, sands with larger permeability coefficients allow for deeper solution infiltration, further influencing the effective reinforcement depth. The influence of the inherent pH of sand on the effective reinforcement depth for low-pH bacterial solution treatment is greater than that of the permeability coefficient. The strength after low-pH bacterial solution treatment ranks as tailings sand > quartz sand > reclaimed sand > calcareous sand. This ranking is primarily determined by the particle size and effective reinforcement depth. Smaller particles result in shallower effective reinforcement depths and higher strengths, with particle size exerting a more significant impact on reinforcement strength than effective reinforcement depth.

At a concentration of 10 × 10^8^ cells/mL, the effective reinforcement depth achieved with the low-pH method is larger compared to the pH-unadjusted method for the same type of sand, and the strength is stronger. This is due to the higher bacterial concentration resulting in sufficient calcium carbonate production, leading to high compactness in both methods and, thus, increasing the strength with effective reinforcement depth. The patterns for sand reinforcement with pH-unadjusted bacterial solution remain consistent with those mentioned earlier. However, the effective reinforcement depth and strength patterns for sand reinforcement with low-pH bacterial solution differ slightly. This is attributed to the high bacterial concentration, which results in insufficient calcium sources in the cementation solution. Therefore, sands like tailings sand and calcareous sand, which can provide additional calcium sources, exhibit greater strength and effective reinforcement depths compared to the other two sands. Furthermore, smaller particle sizes lead to denser reinforcement, resulting in a strength ranking as follows: tailings sand > calcareous sand, with quartz sand > reclaimed sand, in terms of strength.

### 3.6. Wind Erosion Resistance Test

**Figure 12:**
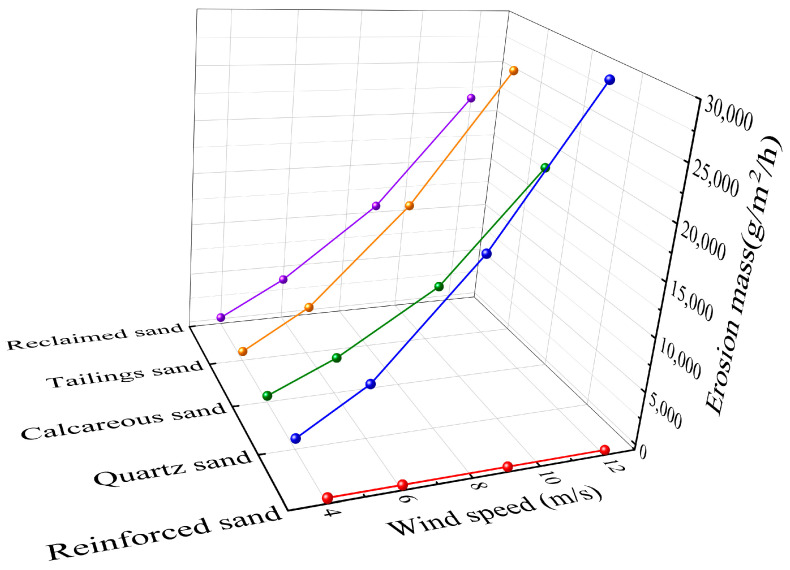
Wind erosion mass.

Figure 12 illustrates the wind erosion mass per unit area for the four types of sand. Prior to reinforcement, the wind erosion mass followed the order: quartz sand > tailings sand > reclaimed sand > calcareous sand. Smaller and lighter sand particles are more susceptible to being carried away by the wind. At a wind speed of 12 m/s, the wind erosion mass of both tailings sand and quartz sand could reach nearly 30 kg/m^2^/h. However, after reinforcement using any method and any concentration, the wind erosion mass for all four types of sand was reduced to zero. This indicates that the MICP-treated sand exhibits exceptional resistance to wind erosion.

## 4. Discussion

### 4.1. Impact of Low pH on Urease Activity

pH value stands as a critical factor affecting microbial life activities, with its impact primarily manifesting in three distinct areas: firstly, by altering the charge of macromolecules within organisms (such as proteins and nucleic acids), thereby affecting their biological activities; secondly, by changing the cell membrane charge, thereby reducing microbial absorption and utilization of nutrients; and thirdly, by diminishing the effectiveness of nutrient utilization in the microbial habitat and enhancing the toxicity of harmful substances [32,33,34,35].

**Figure 13:**
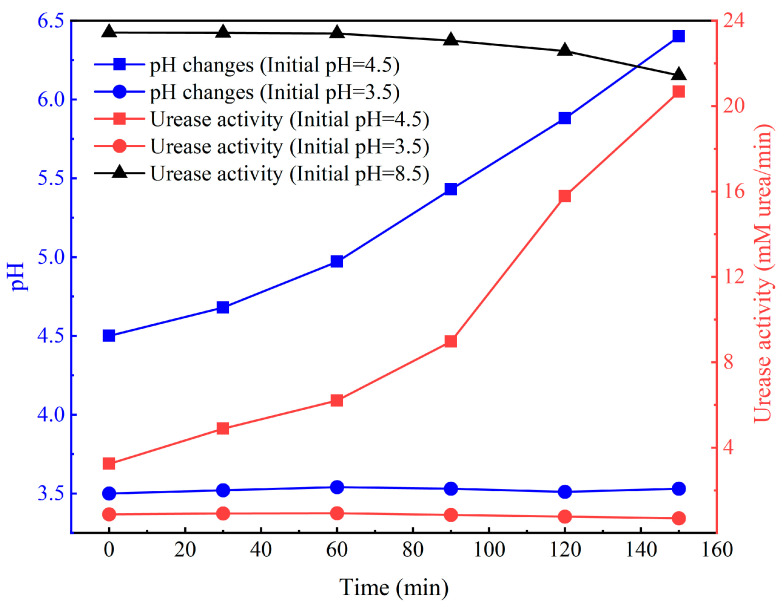
Influence of pH changes on urease activity.

To further investigate the impact of pH variations on urease activity during low-pH MICP reactions, a scenario using a bacterial solution of 0.67 × 10^8^ cells/mL was considered. Two sets of experiments were conducted using bacterial solutions with pH values of 3.5 (below tolerance pH) and 4.5 (minimum tolerance pH) mixed with the cementation solution. The pH of the mixed solution was measured every 30 min, and the results are represented by the blue curve in Figure 13. It is observed that when using an initial pH of 4.5 for the bacterial solution, the pH gradually increased during the reaction process, reaching 5.88 after 2 h and 6.4 after 2.5 h. On the other hand, when using an initial pH of 3.5 for the bacterial solution, the pH remained relatively stable after being mixed with the cementation solution. This observation aligns with the findings in Figure 6 of Section 3.1.

Urease is a protein and functions as an amphoteric electrolyte, making it sensitive to environmental pH. In order to delve deeper into the influence of pH variations on urease activity during low-pH MICP reactions, experiments were conducted using the bacterial solution with an initial pH of 4.5 (0.67 × 10^8^ cells/mL). Sodium hydroxide powder was added every 30 min to adjust the pH of the bacterial solution to match the pH values corresponding to the blue curve in Figure 13. This simulated the pH changes during the MICP reaction process. The conductivity method, outlined in reference [36], was employed to measure urease activity at various time points. A similar approach was taken for the bacterial solution with an initial pH of 3.5 (0.67 × 10^8^ cells/mL) to monitor urease activity. The variation in urease activity for both bacterial solutions over time and pH changes is illustrated by the red curve in Figure 13. For comparison, the bacterial solution’s urease activity at a pH of 8.5 without pH modification (0.67 × 10^8^ cells/mL) is shown by the black curve in Figure 13. One can see that the urease activity of the pH-unadjusted bacterial solution exhibited a slight decrease within 2.5 h but remained relatively high. As for the bacterial solution with an initial pH of 4.5, its urease activity was initially inhibited by the low-pH environment, yielding only 3.24 mM urea/min. The breakdown of urea was limited, leading to slower calcium carbonate precipitation. As the reaction progressed, the pH of the bacterial solution gradually increased, resulting in the recovery of urease activity. At a pH of 5.88 (2 h), the urease activity reached 15.78 mM urea/min, and at a pH of 6.4 (2.5 h), it further increased to 20.67 mM urea/min, nearly reaching the urease activity level of the unadjusted pH condition (21.44 mM urea/min). This is consistent with the conclusion drawn by Whiffin [36] that bacterial urease activity is optimal within the pH range of 6.0 to 8.5. The lag period of the bacterial solution with 0.67 × 10^8^ cells/mL concentration, as shown in Figure 7 of Section 3.1, is 135 min, corroborating the results presented in Figure 13. Finally, for the bacterial solution with an initial pH of 3.5, its urease activity was considerably lower at only 0.88 mM urea/min. The significantly diminished urease activity resulted in insufficient urea breakdown, leading to insufficient OH^−^ production. Additionally, the initial pH (3.5) was too low, and the pH of the bacterial solution could not be rapidly elevated, impeding the rapid recovery of bacterial urease activity. Consequently, under the influence of the low-pH environment, bacterial urease activity gradually diminished, hindering the progress of the MICP process. This observation aligns with the findings presented in Figure 5 of Section 3.1.

To verify the reversibility of urease inactivation, the bacterial solution with an initial pH of 3.5 was used. After 2 h, the pH was adjusted to 8.5 using sodium hydroxide, resulting in a urease activity of 22.67 mM urea/min. This indicates that the temporary inhibition of urease activity due to low pH is reversible. However, if the bacterial solution with an initial pH of 3.5 is adjusted to pH 8.5 after 12 h, the measured urease activity is only 0.32 mM urea/min. This demonstrates that prolonged exposure to a low-pH environment leads to complete urease inactivation.

When employing the low-pH MICP method to reinforce sand, careful consideration must be given to the impact of varying bacterial solution pH on different sand types. For alkaline sands, the bacterial solution pH could be adjusted below the optimal theoretical pH obtained from the beaker tests at the corresponding concentration. Conversely, for acidic soils, the bacterial solution pH should be adjusted above the optimal theoretical pH obtained from the beaker tests at the corresponding concentration. The specific pH values can be determined through preliminary experiments. Sand can be packed into syringe molds, and low-pH bacterial solution can be injected to measure the pH of the effluent. By comparing the difference in pH values, the optimal bacterial solution pH for the specific sand type can be determined. It is essential to note that for acidic soils, the bacterial solution pH must be adjusted above the optimal theoretical pH to prevent further pH reduction upon mixing with the sand. This precaution ensures that the MICP reaction can proceed without hindrance and prevents complete urease inactivation. Additionally, when using low-pH bacterial solution for sand reinforcement, it is important to prepare and use the bacterial solution immediately to avoid prolonged exposure to an acidic environment, which could lead to complete urease inactivation and adversely affect the reinforcement results.

### 4.2. Microscopic Analysis of Different Sands Treated with Low-pH MICP

**Figure 14:**
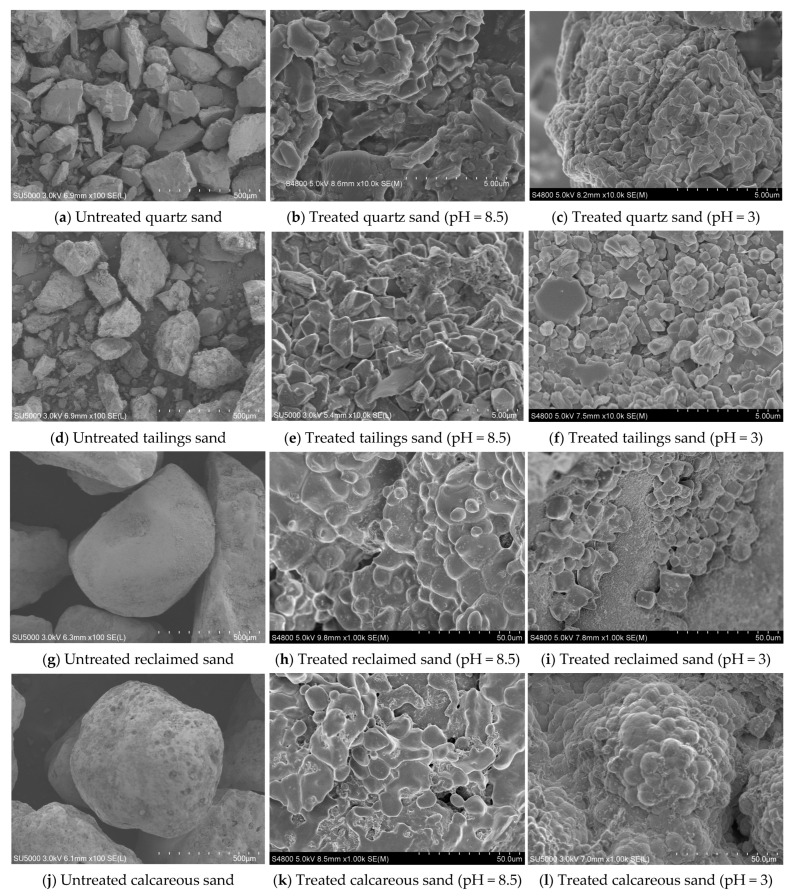
SEM images.

Scanning electron microscopy (SEM) was employed to observe the microscopic structure and morphology of the surfaces of sand samples. Prior to observation, all sand samples were coated with a thin layer of gold to enhance conductivity. Figure 14 presents SEM images of the four types of sand before and after treatment with bacterial solution of 3 × 10^8^ cells/mL concentration. Untreated reclaimed sand and calcareous sand exhibit larger particles compared to tailings sand and quartz sand. The pores between sand particles are also larger, making reinforcement more challenging. After undergoing MICP spray treatment, the initially loose sand particles are bonded together, thereby improving the mechanical properties of the sand.

Spherical calcium carbonate crystals (vaterite) are predominantly generated in calcareous sand and reclaimed sand. These vaterite crystals exhibit an unstable crystal structure and are prone to transformation into aragonite and calcite, and the crystal size is larger. On the other hand, prismatic calcite crystals are mostly formed in quartz sand and tailings sand. These calcite crystals have a more stable crystal structure and smaller crystal sizes and are in higher quantities than those in reclaimed sand and calcareous sand. The abundance of smaller and stable calcite crystals with prismatic shapes facilitates superior pore filling between sand particles, leading to a more stable bonding. Consequently, the overall mechanical properties of tailings sand and quartz sand are superior to those of reclaimed sand and calcareous sand.

For each type of sand, the calcium carbonate induced by urease from non-adjusted pH bacterial solution is generally found scattered or accumulated locally on the surface of sand particles. Conversely, the calcium carbonate induced by urease from low-pH bacterial solution tends to cluster between sand particles, resulting in tighter bonding. As a result, the effectiveness of low-pH MICP reinforcement surpasses that of conventional methods. Notably, the calcite crystals generated in tailings sand are more numerous than those in quartz sand, and they provide a more stable bonding between tailings sand particles compared to quartz sand particles. Among the four types of sand, tailings sand demonstrates the most remarkable reinforcement performance. When treated with a high-concentration bacterial solution under low pH conditions, the reinforced tailings sand exhibits zero erosion due to raindrop impact, indicating exceptional resistance to wind and rain erosion.

In the process of MICP, there exists a competition between the nucleation and growth of calcium carbonate crystals [37]. In sand samples treated with low-pH bacterial solution (pH = 3), the uniform diffusion of bacterial and cementation solutions facilitates the nucleation of new calcium carbonate crystals, while hindering the growth of existing crystals. Conversely, in sand samples treated with non-adjusted pH bacterial solution (pH = 8.5), calcium carbonate tends to precipitate onto existing crystals (growth of crystals) rather than nucleate at new locations. Therefore, in low-pH (pH = 3) MICP-treated sand samples, the size of calcium carbonate crystals is smaller compared to traditional MICP (pH = 8.5).

Furthermore, in sand samples treated with non-adjusted pH bacterial solution (pH = 8.5), calcium carbonate crystals tend to attach to the surface of sand particles or accumulate locally. In contrast, low-pH bacterial solution treatment generates a greater number of calcium carbonate crystals that are uniformly distributed within the sand particles. This can be attributed to the longer flocculation and gelation period at low pH, allowing the bacterial and cementation solutions to penetrate a wider and more even range within the sand particles. As a result, calcium carbonate crystals exhibit a clustered distribution pattern. In summary, the treatment of sand particles with low-pH MICP leads to the strong bonding of sand particles, thereby significantly enhancing the reinforcement effect.

## 5. Conclusions

This study investigated the optimal reaction pH for bacterial solutions with different concentrations, evaluated the reinforcement effects of MICP under the optimal pH on various types of sand, and elucidated the mechanism of low-pH MICP for soil reinforcement. The main conclusions are as follows:

(1) Low pH can delay the flocculation of calcium carbonate. Within the tolerated pH range, lower bacterial solution pH leads to more calcium carbonate generation and a longer bioflocculation lag period. Higher bacterial solution concentration results in more calcium carbonate production but a shorter bioflocculation lag period. The optimal theoretical reaction pH values for bacterial solution concentrations of 0.67 × 10^8^, 3 × 10^8^, and 10 × 10^8^ cells/mL are 4.5, 3, and 4, respectively;

(2) For the same type of sand, using low-pH bacterial solutions yields better reinforcement results than traditional methods of unadjusted pH. The bioflocculation lag periods of 0.67 × 10^8^ (pH = 4.5), 3 × 10^8^ (pH = 3), and 10 × 10^8^ (pH = 4) cells/mL bacterial solutions are 540, 55, and 45 min. And the shorter bioflocculation lag period leads to a lower effective reinforcement depth but a higher reinforcement strength;

(3) With 0.67 × 10^8^, 3 × 10^8^, and 10 × 10^8^ cells/mL bacterial solutions, the strength of tailings sand containing calcium salt was 21.15%, 44.42%, and 13.61% higher than quartz sand, respectively. The effective reinforcement depth of alkaline reclaimed sand was 10, 8, and 6 mm lower than that of neutral calcareous sand, respectively. The strength of fine tailings sand was 70.41%, 58.04%, and 22.6% higher than that of coarse reclaimed sand. The effective reinforcement depth of fine quartz sand was 6, 4, and 4 mm lower than that of coarse calcareous sand;

(4) The primary mechanism of low-pH MICP reinforcement lies in the transient inhibition of bacterial urease activity due to low pH. This inhibition delays calcium carbonate precipitation. During this period, bacterial and cementation solutions penetrate the soil uniformly. As bacterial solution pH increases, urease activity gradually recovers, leading to the uniform filling of inter-particle voids in sand with calcium carbonate crystals. This results in improved mechanical performance and enhanced uniformity of reinforcement;

(5) When applying low-pH MICP to soil reinforcement, adjusting the optimal reaction pH values for various bacterial solution concentrations based on soil properties is essential. Additionally, bacterial solutions should be freshly prepared and used promptly to prevent urease inactivation and subsequent reinforcement failure.

In general, we explored the influences of bacterial solution concentration and pH on calcium carbonate production and bioflocculation lag period and utilized the bioflocculation lag period to improve the uniformity of reinforcement. The optimal pH value of different sands reinforced by MICP at low pH values was obtained. We also explored the mechanism of urease inactivation and reversible recovery at low pH values and revealed the mechanism of MICP reinforcement of sands at low pH.

## Figures and Tables

**Table 1:** Basic properties and main components of test sands.

Category	Tailings Sand	Reclaimed Sand	Calcareous Sand	Quartz Sand
Type	fine sand	coarse sand	coarse sand	fine sand
pH	9.5	9.8	7.8	7.2
Main components	SiO_2_, CaO	SiO_2_	SiO_2_, CaO	SiO_2_
*ρ*_dmin_ (g/cm^3^)	1.8283	1.3289	1.3360	1.3378
*ρ*_dmax_ (g/cm^3^)	2.3746	1.6387	1.6713	1.9405

## Data Availability

Data are not publicly available, although the data may be made available on request from the corresponding author.
